# Biallelic *KIF24* Variants Are Responsible for a Spectrum of Skeletal Disorders Ranging From Lethal Skeletal Ciliopathy to Severe Acromesomelic Dysplasia

**DOI:** 10.1002/jbmr.4639

**Published:** 2022-07-19

**Authors:** Madeline Louise Reilly, Noor ul Ain, Mari Muurinen, Alice Tata, Céline Huber, Marleen Simon, Tayyaba Ishaq, Nick Shaw, Salla Rusanen, Minna Pekkinen, Wolfgang Högler, Maarten F. C. M. Knapen, Myrthe van den Born, Sophie Saunier, Sadaf Naz, Valérie Cormier‐Daire, Alexandre Benmerah, Outi Makitie

**Affiliations:** ^1^ Imagine Institute, Laboratory of Inherited Kidney Diseases, Institut National de la Santé et de la Recherche Médicale (INSERM) Unités Mixtes de Recherche (UMR) 1163 Université Paris Cité Paris France; ^2^ School of Biological Sciences University of the Punjab Lahore Pakistan; ^3^ Department of Molecular Medicine and Surgery and Center for Molecular Medicine Karolinska Institutet Stockholm Sweden; ^4^ Folkhälsan Research Center Helsinki Finland; ^5^ Children's Hospital University of Helsinki and Helsinki University Hospital Helsinki Finland; ^6^ Research Program for Clinical and Molecular Metabolism University of Helsinki Helsinki Finland; ^7^ Imagine Institute, Laboratory of Molecular and Physiopathological bases of Osteochondrodysplasia, Institut National de la Santé et de la Recherche Médicale (INSERM) Unités Mixtes de Recherche (UMR) 1163 Université Paris Cité Paris France; ^8^ Department of Genetics, Reference Centre for Skeletal Dysplasia, Assistance Publique–Hôpitaux de Paris Necker‐Enfants Malades Hospital Paris France; ^9^ Department of Medical Genetics University Medical Centre Utrecht Utrecht The Netherlands; ^10^ Department of Endocrinology & Diabetes Birmingham Children's Hospital Birmingham UK; ^11^ Institute of Metabolism and Systems Research University of Birmingham Birmingham UK; ^12^ Department of Paediatrics and Adolescent Medicine Johannes Kepler University Linz Linz Austria; ^13^ Department of Obstetrics and Fetal Medicine Erasmus Medical Center Rotterdam The Netherlands; ^14^ Department of Clinical Genetics, Erasmus MC University Medical Center Rotterdam The Netherlands; ^15^ Present address: Madeline Louise Reilly, Department of Neuroscience, School of Life Sciences University of Sussex UK; ^16^ Present address: Noor ul Ain Institute of Biomedical and Genetic Engineering Islamabad Pakistan

**Keywords:** ACROMESOMELIC DYSPLASIA, SKELETAL DYSPLASIA, PRIMARY CILIA, CILIOPATHIES, KINESIN

## Abstract

Skeletal dysplasias comprise a large spectrum of mostly monogenic disorders affecting bone growth, patterning, and homeostasis, and ranging in severity from lethal to mild phenotypes. This study aimed to underpin the genetic cause of skeletal dysplasia in three unrelated families with variable skeletal manifestations. The six affected individuals from three families had severe short stature with extreme shortening of forelimbs, short long‐bones, and metatarsals, and brachydactyly (family 1); mild short stature, platyspondyly, and metaphyseal irregularities (family 2); or a prenatally lethal skeletal dysplasia with kidney features suggestive of a ciliopathy (family 3). Genetic studies by whole genome, whole exome, and ciliome panel sequencing identified in all affected individuals biallelic missense variants in *KIF24*, which encodes a kinesin family member controlling ciliogenesis. In families 1 and 3, with the more severe phenotype, the affected subjects harbored homozygous variants (c.1457A>G; p.(Ile486Val) and c.1565A>G; p.(Asn522Ser), respectively) in the motor domain which plays a crucial role in KIF24 function. In family 2, compound heterozygous variants (c.1697C>T; p.(Ser566Phe)/c.1811C>T; p.(Thr604Met)) were found C‐terminal to the motor domain, in agreement with a genotype–phenotype correlation. In vitro experiments performed on amnioblasts of one affected fetus from family 3 showed that primary cilia assembly was severely impaired, and that cytokinesis was also affected. In conclusion, our study describes novel forms of skeletal dysplasia associated with biallelic variants in *KIF24*. To our knowledge this is the first report implicating *KIF24* variants as the cause of a skeletal dysplasia, thereby extending the genetic heterogeneity and the phenotypic spectrum of rare bone disorders and underscoring the wide range of monogenetic skeletal ciliopathies. © 2022 The Authors. *Journal of Bone and Mineral Research* published by Wiley Periodicals LLC on behalf of American Society for Bone and Mineral Research (ASBMR).

## Introduction

Skeletal dysplasias are a group of inherited disorders which are both clinically and genetically heterogeneous. More than 430 genes have been identified to cause approximately 460 different skeletal disorders, whereas genetic etiology for many of these rare conditions is still unknown.^(^ ^1^ ^)^ The latest nosology of genetic skeletal disorders published in 2019 remains hybrid in nature, with some diseases grouped based on the causal gene while others are grouped based on radiological or clinical features. For example, mesomelic dysplasia (group 17) are characterized by shortening of the middle segments of the limbs. On the other hand, skeletal ciliopathies (group 9) vary in skeletal features but share a common etiology, namely defective function of primary cilia (PC). PC are sensory antennae, found at the surface of most cell types, that control key signaling pathways during development and/or tissue homeostasis. PCs are involved in the sensing of light (photoreceptor, retina), odors (olfactory cilia), and flow (kidney tubules), as well as in the control of a still increasing list of signaling pathways (Hedgehog, transforming growth factor β [TGF‐β], G protein‐coupled receptors, etc.). Ciliopathies caused by PC dysfunction include a wide range of isolated or syndromic forms affecting diverse organs or tissues including the retina, kidney, and brain.^(^ ^2^, ^3^ ^)^

Primary cilia are formed in quiescent cells or cells undergoing terminal differentiation. During this process, the mother centriole of the centrosome docks onto cellular membranes and then elongates to form the axoneme composed of nine doublets of microtubules. This initial step of ciliogenesis is controlled by complex cell cycle–regulated events in which CP110 and its partners, including the microtubule depolymerizing kinesin KIF24, play a crucial role.^(^ ^4^ ^)^ The ciliary membrane around the axoneme is highly enriched for specific signaling transmembrane proteins and its composition is maintained by a filter‐like region called the transition zone, present at the base of the cilium. Ciliary components, including signaling intermediates, are imported through the transition zone by an active process based on the intraflagellar transport (IFT) machinery, which is composed of two subcomplexes, IFT‐B and IFT‐A, involved in anterograde (import) and retrograde (IFT recycling, export) transport, respectively. IFT is dependent on microtubule‐based motors including Kinesin‐II (anterograde) and cytoplasmic dynein 2 (retrograde) and is required for ciliogenesis. Loss of function of most IFT components results in severe ciliogenesis defects (or very short stunted cilia for retrograde IFT) as well as in very early and severe developmental defects in vertebrates. However, hypomorphic mutations of IFT‐encoding genes are a common cause of ciliopathies in humans, mostly affecting the skeleton.^(^ ^2^, ^3^ ^)^

Skeletal ciliopathies mainly refer to the autosomal recessive group of short‐rib polydactyly syndromes (SRPSs) with or without polydactyly. SRPSs are characterized by narrow thorax, trident acetabular roof, occasional polydactyly, and limb shortening and bowing associated with possible anomalies of major organs such as the brain, eye, heart, kidneys, liver, pancreas, intestines, and genitalia.^(^ ^1^ ^)^ SRPS encompasses Ellis‐van Creveld syndrome (EVC; MIM 225500), Jeune syndrome or asphyxiating thoracic dystrophy (ATD; MIM 208500), both compatible with life; SRP1–SRP4, which are all lethal conditions, including Verma‐Naumoff syndrome (SRP type III [SRP3]; MIM 613091), Sensenbrenner syndrome (MIM 218330), and Mainzer‐Saldino syndrome (MZSDS; MIM 266920). There is phenotypic overlap between the various forms of SRPS but the conditions also differ by multiorgan involvement, metaphyseal appearance, and prognosis.

The ATD spectrum is genetically heterogeneous and linked to variants in IFT genes affecting the PC. Variants in more than 16 different genes have been identified in ATD: *IFT52*, *IFT172*, *IFT80*, *IFT81*, and *TRAF3IP1* (involved in anterograde intraflagellar transport [IFT‐B]); *IFT140*, *WDR35*, *WDR19*, and *TTC21B* (retrograde intraflagellar transport [IFT‐A]); *DYNC2H1*, *DYNC2LI1*, *WDR34*, *TCTEX1D2*, and *WDR60* (retrograde intraflagellar transport, cytoplasmic dynein motor); and *CEP120* and *TALPID3* (centrosomal proteins).^(^ ^1^ ^)^ More recently variants of *CFAP410* and *NEK1* have been reported in axial spondylometaphyseal dysplasia, characterized by rhizomelic short stature, platyspondyly, and retinal changes, further expanding the phenotypic variability of skeletal ciliopathies.^(^ ^5^, ^6^ ^)^

In the present study, we investigated individuals in three families with unknown skeletal dysplasia. In one of the families, three fetuses presented a form of skeletal ciliopathy resembling ATD. We also analyzed two individuals from a family affected by a novel form of acromesomelic dysplasia and one individual from a third unrelated family presenting a milder skeletal dysplasia with platyspondyly. By performing whole‐genome, whole‐exome, or targeted exome sequencing, we identified biallelic missense variants in *KIF24* as the likely cause of their disorder. We also provide functional evidence that ciliogenesis and cytokinesis are severely affected in fibroblasts from one of the affected fetuses with *KIF24*‐related SRPS. This is the first report implicating *KIF24* variants in any disorder.

## Subjects and Methods

### Ethics statements

This study was approved by the institutional review board (IRB) of School of Biological Sciences, University of the Punjab, Lahore, Pakistan (Ethics approval SBS11‐1 by IRB 00005281); the Research Ethics Committee of the Helsinki University Central Hospital, Finland; the institutional review board of Necker Hospital (IRB 00011928, 2020‐04‐06); and the Research Ethics Committee of the University Medical Center, Rotterdam, The Netherlands. Written informed consent was obtained from all participants, or the guardians in case of minors. All subjects were recruited to the study as part of research projects aiming to identify novel disease‐causing genes and gene variants in individuals with skeletal dysplasia; their clinical care and follow up were not part of the study but occurred according to standard practices. Appropriate informed consents were obtained from patients or their guardians prior to inclusion to the study.

### Subjects

**Fig. 1:**
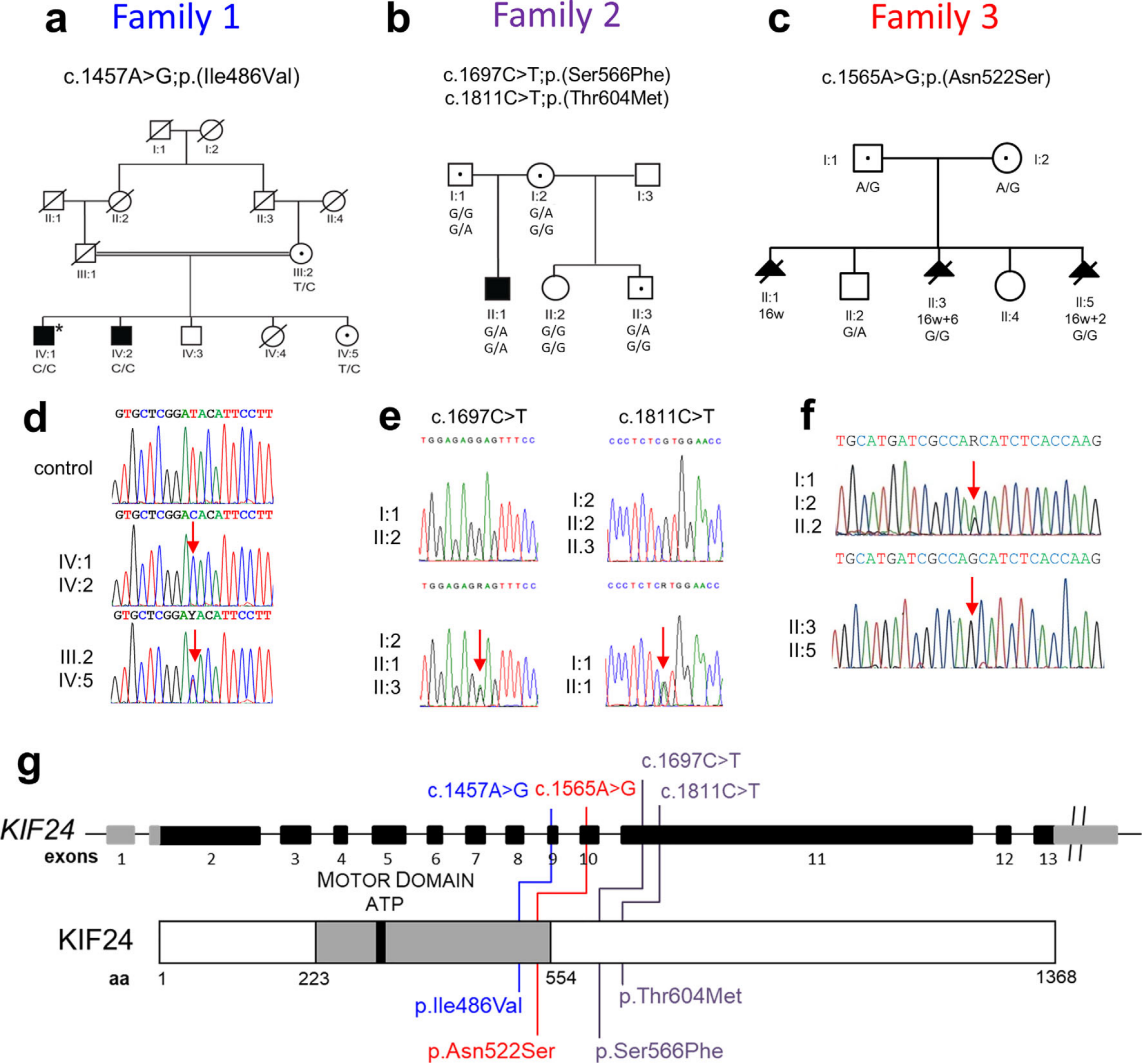
Identification of missense variants in *KIF24* in skeletal dysplasia. (*A*–*C*) Pedigrees of the identified families (1 to 3). Filled symbols represent affected individuals and double line indicates consanguinity. Genotypes of identified variants are given below symbols of individuals whose DNA samples were available for Sanger sequencing. (*D*–*F*) Verification of the identified variants by Sanger sequencing in the three families. Variant nucleotide in each of the partial chromatogram of DNA sequence is depicted by a red arrow. (*G*) Schematic representation of *KIF24* exons and protein organization (motor domain) showing the position of the identified variants and corresponding amino acid variations.

Family 1 (TID‐01) was a consanguineous Pakistani family from the Punjab province with two affected individuals and two unaffected siblings (Fig. 1*A*). Clinical history was obtained for onset and progression of the disorder. Heights of all participating individuals were measured. Photographs of arms, legs, and hands, and radiographs of forearms, forelegs, and spine of one affected individual were taken for clinical evaluation. Blood samples were drawn from all participating individuals. DNA was isolated by sucrose lysis and salting out method.

Family 2 was recruited from the Birmingham Children's Hospital, Birmingham, UK. The family comprised unrelated white parents with one affected child. The child has two unaffected maternal half‐siblings (Fig. 1*B*). Clinical and family histories were obtained. Radiographs of arms, legs, hands, and spine were available for evaluation. Blood parameters of bone mineral homeostasis were measured, and a transiliac bone biopsy was obtained. Blood samples were drawn from all participating family members and DNA was extracted using standard procedures.

Family 3 was recruited from the Erasmus MC, Rotterdam, The Netherlands. Parents were both white and unaware of consanguinity; they have one healthy son and one healthy daughter. Three pregnancies were terminated because of severe fetal growth restriction and skeletal abnormalities resembling ATD (Fig. 1*C*). Radiographs of the affected fetuses were available for evaluation. DNA was isolated from chorionic tissue, whole blood, and/or umbilical cord tissue. Blood samples were drawn from the parents and the oldest child and DNA was extracted using standard procedures.

### Molecular analysis

Whole‐genome sequencing (WGS) was performed with average coverage of 30× for index patient IV:1 of family 1 as described^(^ ^7^ ^)^ at the Science for Life Laboratory (SciLife Lab), Stockholm, Sweden. Annotation of variants was performed using Variant Effect Predictor (VEP)^(^ ^8^ ^)^ and then uploaded into a database generated by GEMINI.^(^ ^9^ ^)^ Annotated variants were filtered as described.^(^ ^7^ ^)^ Filtered variants were further evaluated based on the conservation (genomic evolutionary rate profiling [GERP]) and pathogenicity scores (combined annotation‐dependent depletion [CADD]). Sanger sequencing was performed to examine segregation of the variant with the skeletal phenotype in all family members as well its absence from 200 ethnically matched controls from Pakistan.

In family 2, whole‐exome sequencing (WES), including all protein coding exons and exon‐intron boundaries (±20 base pairs [bp]), was performed for the patient and his parents. Paired‐end sequencing was performed using the Illumina sequencing system (Illumina, San Diego, CA, USA) at Blueprint Genetics, Helsinki, Finland. Median sequencing coverage was 269 for the index patient, 280 for the mother and 221 for the father. Reads were mapped to the human reference genome (GRCh37/hg19). Variants were annotated and filtered using the VarAFT tool (https://varaft.eu). Frequency data for initial filtering was obtained from the Genome Aggregation Database, the 1000 Genomes Project, Kaviar, and the Haplotype Reference Consortium integrated in the VarAFT tool. After initial filtering, further variant frequency data was obtained from dbSNP (https://www.ncbi.nlm.nih.gov/snp/). The variants were observed in the Integrative Genomics Viewer (IGV) and further evaluated based on literature search and pathogenicity scores. Sanger sequencing was performed to examine segregation of candidate variants with the affected status in all family members. The family's WES data was analyzed for copy number variations (CNVs) with four programs, CoNIFER (version 0.2.2), XHMM (v 1.1), ExomeDepth (v 1.1.15), and CODEX (v 1.18.0). The detection results were combined with a minimum overlap requirement of 1 bp, and then filtered with a logistic regression model (R 3.6.3; R Foundation for Statistical Computing, Vienna, Austria; https://www.r-project.org/) for predicted true‐positive results. This pipeline has been described in detail.^(^ ^10^ ^)^ The results were annotated with an in‐house upgraded version of cnvScan^(^ ^11^ ^)^ with databases of common CNVs from 1000 Genomes, DGV, DECIPHER, ExAC, and gnomAD (v 2.1) and an in‐house CNV database from previously analyzed WES samples (*n* = 268). We looked for de novo CNVs, which were present in these databases with a minor allele frequency (MAF) of <1% with 50% and 90% reciprocal overlap requirements.

To identify the genetic variants responsible for ATD in family 3, we subjected the genomic DNA of an affected fetus born to unrelated parents to ciliome sequencing, using a 5.3‐megabase (Mb) customized Agilent SureSelect Target Enrichment library to capture 32,146 exons of 1666 genes.^(^ ^12^, ^13^ ^)^ We first focused our analysis on consensus splice‐site changes, nonsynonymous variants, and insertions and/or deletions in the coding regions. Considering that ATD‐causing variants are rare, we assumed that the affected individual was likely homozygous for variants absent in the dbSNP132, 1000Genome, and in‐house databases. Sanger sequencing was performed to examine the segregation of the variant with the ATD phenotype.

### Cell culture

Fetal‐derived amniocytes from control (CT#1; a kind gift from Sophie Thomas, Institut Imagine, France) or from the second fetus in family 3 (F22) were cultured in AmnioMAX™‐II Complete Medium (Thermo Fisher Scientific, Cramlington, UK; 11269016). Control fibroblasts (CT#2; Promocell, Heidelberg, Germany; C‐12300) and those derived from an unrelated case of renal hypodysplasia (CT#3; a kind gift from Cecile Jeanpierre, Institut Imagine, France) were cultured in Opti‐MEM medium (Invitrogen, Thermo Fisher Scientific) supplemented with 10% fetal bovine serum (FBS), penicillin/streptomycin and glutamine (all Invitrogen, Thermo Fisher Scientific). In each case, culture surfaces were coated with collagen I, Rat Tail (Corning, Inc., Corning, NY, USA; 354236). Ciliogenesis was induced by 24 hours of serum starvation in Opti‐MEM medium.

### Antibodies and immunofluorescence

The primary antibodies used were mouse anti‐centrin (1:400; Sigma‐Aldrich, St. Louis, MO, USA; clone 20H5), rabbit anti‐Arl13b (1:200; Proteintech Group, Inc., Rosemont, IL, USA; 17711‐1‐AP), mouse anti‐GT335 (1:2000; Adipogen AG, Fuellinsdorf, Switzerland; AG‐20B‐0020), human anti‐ninein (1:200, a gift from James Sillibourne, Curie Institute), mouse anti‐α‐tubulin (1:1000; Abcam, Cambridge, UK; ab18251), goat anti‐γ‐tubulin (1:200; Santa Cruz Biotechnology, Santa Cruz, CA, USA; sc‐7396). Cells were incubated with secondary antibodies conjugated to AlexaFluor® 488, 555 or 647 (1:400; Molecular Probes, Thermo Fisher Scientific; donkey).

Cells were cultured on cover slips and fixed in either 4% paraformaldehyde on ice or, for microtubule staining, in methanol at room temperature. Incubation of primary and secondary antibodies was performed in Dulbecco's phosphate buffered saline (PBS; Sigma‐Aldrich), 0.1% Triton X‐100 (Sigma‐Aldrich), and 1 mg/mL or 3% bovine serum albumin (BSA; Sigma‐Aldrich). Cells were washed three times between primary and secondary antibody incubation, followed by three washes in PBS. Nuclear staining was performed using 4′,6‐diamidino‐2‐phenylindole (DAPI, Sigma‐Aldrich; MBD0015). Cover slips were mounted onto glass slides using Mowiol® 4‐88 (Sigma‐Aldrich). Stained cells were then imaged using an epi‐illumination microscope (DMR; Leica, Wetzlar, Germany) with a cooled charge‐coupled device camera (Leica; DFC3000G). Images were acquired with LAS (Leica V4.6) and processed with ImageJ software (NIH, Bethesda, MD, USA; https://imagej.nih.gov/ij/) and Photoshop CS2 (Adobe Systems, Inc., San Jose, CA, USA).

## Results

### Clinical and skeletal defects in affected individuals and fetuses

#### Family 1

**Fig. 2:**
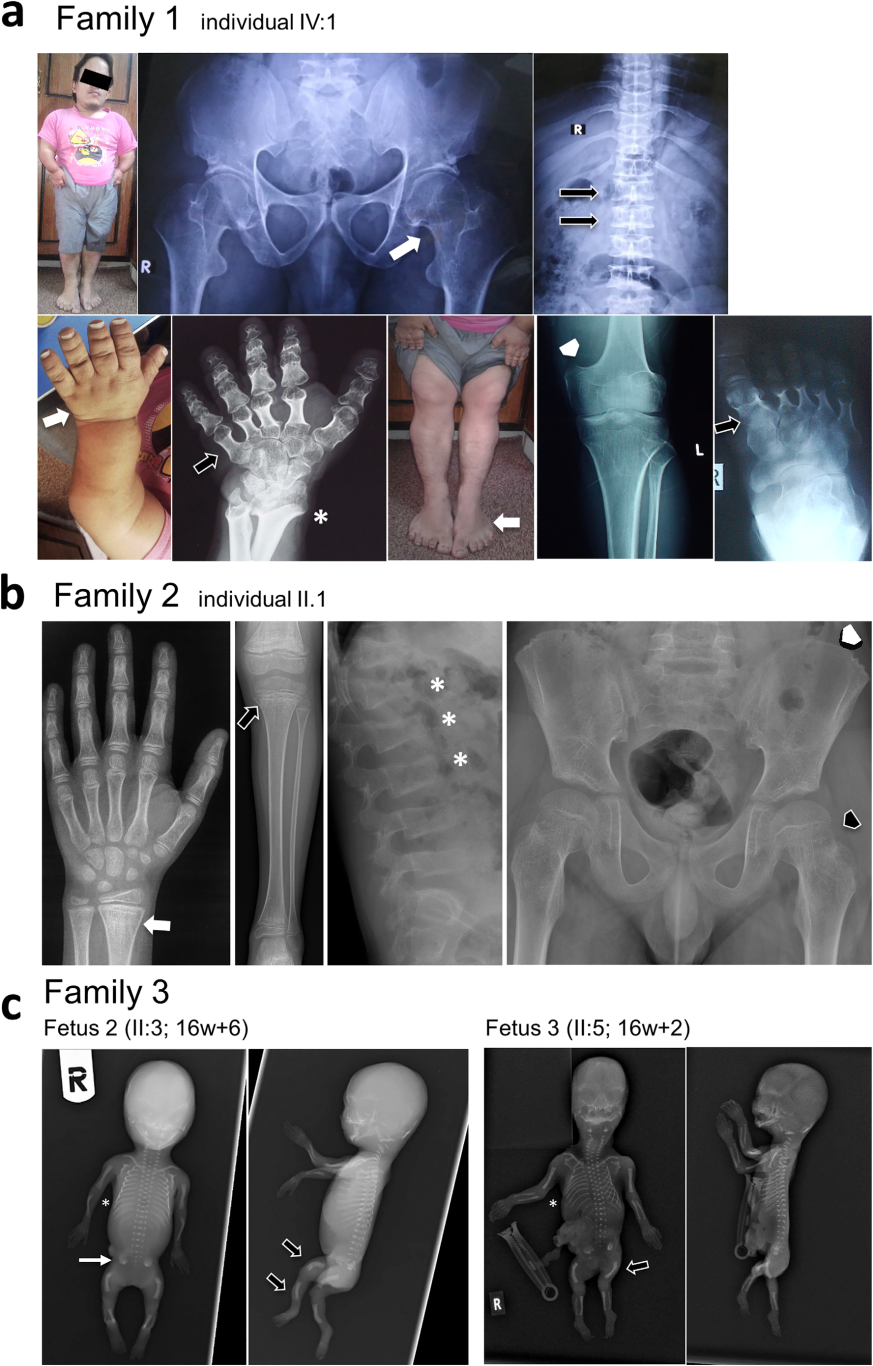
Clinical phenotype in the affected participants. (*A*) Photographs and radiographs of individual IV:1 from family 1. Photograph shows disproportionate short stature with acromesomelic shortening of forelimbs and facial hypoplasia with broad forehead. Hands and feet are short and broad on photographs (lower panel, white arrows) and radiographs show extremely short tubular bones (black arrows) and abnormal distal radius and ulna (white asterisk). Pelvic radiograph shows short and broad femoral necks (upper panel, white arrow). Radiograph of the spine shows platyspondyly (upper panel, black arrows). Knee radiograph indicates wide metaphyses (white arrowhead). (*B*) Radiographs of the affected individual from family 2. Radiograph of the hand shows osteopenia and an irregular mineralization pattern at metaphyses (white arrow). Radiograph of the lower limb shows osteopenia and metaphyseal irregularities (black arrow). Radiographs of the spine indicate significant osteopenia and platyspondyly (white asterisks). Pelvic radiograph shows in addition to generalized osteopenia, mild coxa valga with short and broad femoral necks (black arrowhead) and mild irregularity in the iliac wing (white arrowhead). (*C*) Radiographs of two of the affected fetuses from family 3 (fetus 2 [F22], 16 weeks’ gestation +6; fetus 3, 16 weeks’ gestation +2). Note in both fetuses the small round ilium with trident acetabulum (white arrow) and shortening and bowing of the long tubular bones (black arrows). Thorax appeared slightly narrow (white asterisks).

Family 1 had two affected brothers born to consanguineous parents (Fig. 1*A*). The father was deceased and both brothers were adults at the time of recruitment of the family. The affected individuals had acromesomelic‐like skeletal dysplasia with severe shortening of forelimbs and extreme short stature. Their adult heights were 111 cm and 121 cm, corresponding to −9.1 and −7.7 standard deviation (SD), respectively. They had abnormal gait and restricted joint movements accompanied by pain. Cognition was grossly normal, and both were gainfully employed. There were no clinical symptoms suggestive of impaired immunity. Radiographs of the affected individual IV:1 indicated several skeletal anomalies (Fig. 2*A*). Radiographs of hand and feet showed severely shortened metacarpals and metatarsals. Humeri were also short. Spinal radiographs revealed platyspondyly involving the whole spine. Pelvic radiograph indicated hip dysplasia with deformed proximal femurs and short femoral neck. Femurs were short with widening of the metaphyseal regions around the knees.

#### Family 2

The index patient of family 2 was an 11‐year‐old white boy, born to nonconsanguineous parents (Fig. 1*B*), who presented with knee pains and limping at the age of 7 years. He was initially thought to have juvenile arthritis but showed no response to treatment. At the age of 8 years his mobility started to deteriorate. He was able to walk only short distances and required the use of a wheelchair. He had short stature (−2.6 SD) and a slow growth velocity of 2.2 cm per year. Spinal radiographs revealed reduced vertebral height suggesting compression fractures. Long‐bone radiographs showed irregularities in the metaphyseal regions. In pelvic radiograph, a mild irregularity was observed in the iliac wing (Fig. 2*B*). At the age of 9 years, he had low bone density at the lumbar spine (*Z*‐score −2.7) and whole body (*Z*‐score −2.8). Genetic testing for osteogenesis imperfecta was negative. He had normal dentition, white sclerae, and no hypermobility. Bone biopsy revealed cortical and trabecular osteopenia with markedly increased cortical turnover. There were unusual foci of callus‐like new bone formation on some endosteal surfaces. His plasma calcium, phosphate, and alkaline phosphatase were normal. Level of parathyroid hormone was slightly elevated (44 ng/L). None of his family members had a history of recurrent fractures or osteoporosis.

#### Family 3

Parents from Family 3 were white, self‐declared as nonconsanguineous, and they had two healthy children and three affected fetuses (Fig. 1*C*). Fetus 2 (II:3; F22) presented some features of ATD including a small round ilium with trident acetabulum and shortening and bowing of the long tubular bones (Fig. 2*C*), together with kidney microcysts and fibrosis. The ribs were not too short, but thorax appeared slightly narrow. This pattern was very similar to that observed in two other fetuses from two independent pregnancies except regarding the kidneys, which appeared macroscopically normal for fetus 3 (no information for fetus 1). These anomalies as well as the fetal growth restriction and oligoamnios/anhydramnios led to the termination of the pregnancies for all three affected fetuses between gestational weeks 16 and 17.

### Identification of biallelic missense *KIF24* variants

The phenotype in family 1 resembled that of acromesomelic dysplasia Maroteaux type (MIM 602875). However, linkage to *NPR2* was not observed (data not shown). Analysis of WGS data of individual IV:1 shortlisted 12 possible pathogenic variants with MAF <0.01 (Table S1). Further filtration of variants highlighted two likely pathogenic variants in *FMN1* and in *KIF24*. No other potential pathogenic variant was identified in the genome sequencing data that could account for the disease phenotype in this family. Only the homozygous missense variant c.1456A>G; p.(Ile486Val) in *KIF24* (NM_194313.2) segregated with the disease phenotype. Both affected individuals were homozygous for this variant whereas their mother and an unaffected sister were heterozygous (Fig. 1*A*,*D*). In addition, the identified variant was extremely rare in all databases (gnomAD allele frequency 0.00003630, none homozygous: *rs370470782*) and was absent in Sanger sequenced DNA of 200 ethnically matched controls (400 chromosomes). This variant was predicted to be damaging by various mutations prediction tools including MutationTaster and polymorphism phenotyping2 (PolyPhen2) with very high CADD and REVEL scores. The Ile486 residue was absolutely conserved in all 100 vertebrate species examined (https://genome-euro.ucsc.edu, https://www.ncbi.nlm.nih.gov/homologene?cmd=Retrieve&dopt=MultipleAlignment&list_uids=52346).

For family 2, CNV analysis using WES data of the affected individual (II:1) and his parents was performed. The analysis was negative for any known disease‐causing variants that could explain the patient's phenotype. Additionally, the WES data was analyzed for rare CNVs, but no de novo rare CNVs that could explain the patient's phenotype were detected. In analysis of WES data, de novo single‐nucleotide variant (SNV) and indel variants with up to 0.001 MAF, homozygous or compound heterozygous variants with up to 0.015 MAFs, and X‐chromosomal variants with up to 0.002 MAFs were considered. Intronic variants, intergenic variants, and synonymous variants were not considered in the analysis. After filtering, and reviewing the variants in IGV, there were no homozygous variants, two de novo variants, two X‐chromosomal variants, and six compound heterozygous variants (Table S2). After a literature search, evaluation of variants’ pathogenicity scores, conservation, biological function of the encoded protein, and segregation of the variants in the family (Fig. 1*B*,*E*), we considered the compound heterozygous *KIF24* (NM_194313.2) variants as the most likely to be causative for the affected individual's phenotype. The heterozygous missense variant c.1811C>T; p.(Thr604Met) in *KIF24* was inherited from the father with a MAF of 0.000211 in gnomAD exomes (*rs201469275*), and was predicted damaging by protein variation effect analyzer (PROVEAN). The heterozygous missense variant c.1697C>T; p.(Ser566Phe) in *KIF24* was inherited from the mother, with a MAF of 0.00579 in gnomAD exomes (r*s139062260*), and was predicted pathogenic by DANN (https://swmath.org/software/31340), LIST‐S2 (https://list-s2.msl.ubc.ca/), and Mutation assessor (http://mutationassessor.org/r3/). The two variants affected amino acids which were not conserved in evolution (https://www.ncbi.nlm.nih.gov/homologene?cmd=Retrieve&dopt=MultipleAlignment&list_uids=52346). However, the affected individual was the only one in the family who had both variants.

The observed manifestations in the affected fetus 2 from family 3 (skeletal dysplasia and cystic kidneys) were indicative of a skeletal ciliopathy resembling ATD. Biallelic damaging variations could not be identified after sequencing the coding sequence of *DYNC2H1*, the main cause of SRP type III.^(^ ^1^ ^)^ By a larger “ciliome” panel analysis, we identified a homozygous missense variant c.1565A>G; p.(Asn522Ser) *rs535585536* in *KIF24* (NM_194313.2). This variant has a frequency of 0.0001026 in gnomAD and was predicted damaging by sorting intolerant from tolerant (SIFT) and PolyPhen. Segregation of the identified missense variant was performed by Sanger sequencing, which showed that both parents as well as an unaffected sibling were heterozygous for the identified variant whereas fetus 2 and fetus 3 were homozygous (Fig. 1*C*,*F*). The identified variant affects an Asn residue encoded by exon 9 (Fig. 1*G*), which is present in the motor domain of KIF24 within a highly conserved stretch of amino acids (100% conservation between human and diverse vertebrate species including *Xenopus laevis*; https://www.ncbi.nlm.nih.gov/homologene?cmd=Retrieve&dopt=MultipleAlignment&list_uids=52346).

### The homozygous p.(Asn522Ser) *KIF24* variant identified in fetuses leads to defective ciliogenesis and cytokinesis in amnioblasts

**Fig. 3:**
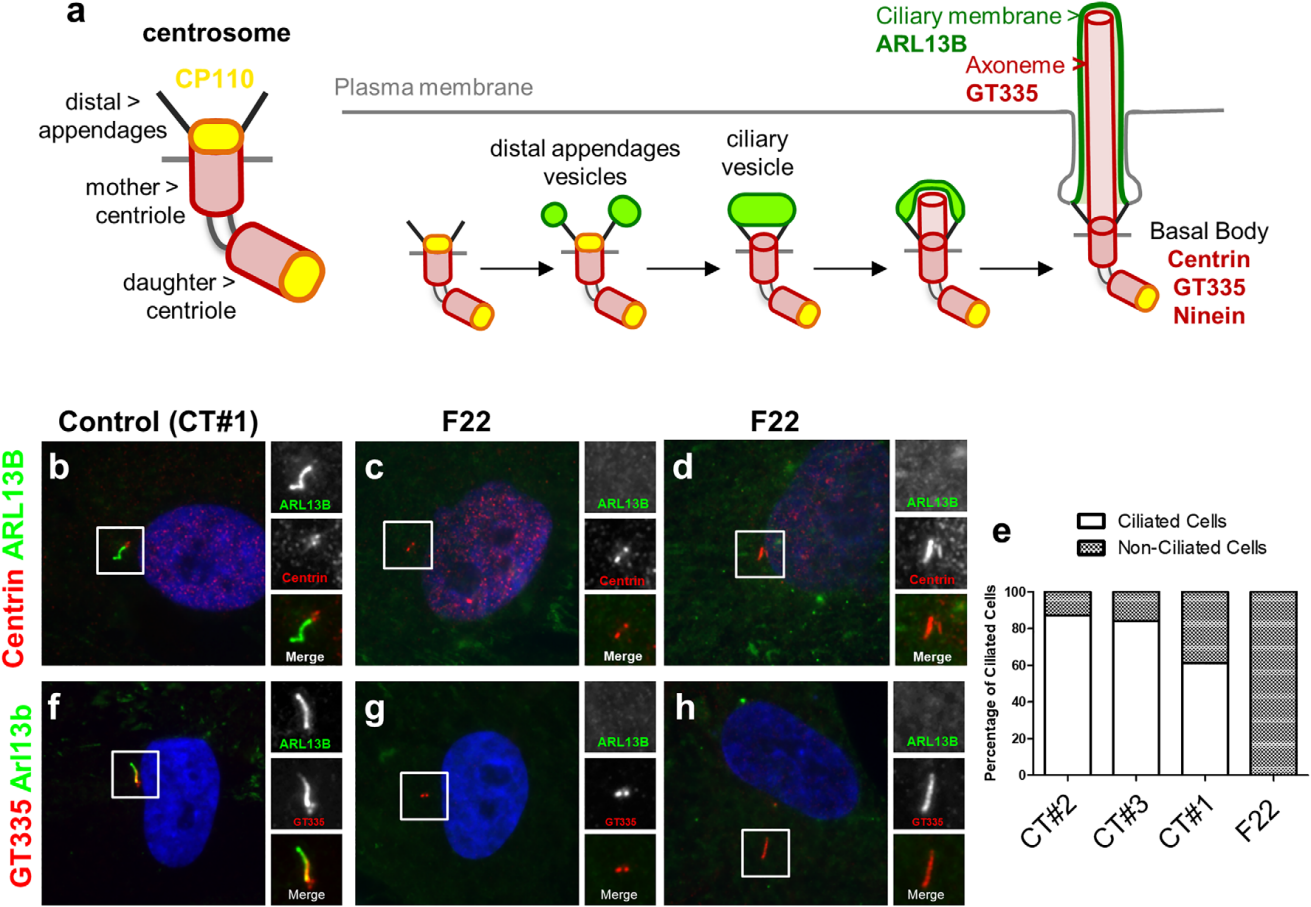
Ciliogenesis is severely affected in fibroblasts from one of the SRPS fetuses. (*A*) Schematic representation of ciliogenesis in fibroblasts where the main markers of ciliary subcompartments are indicated. Control (CT#1, CT#2, and CT#3) and F22 cells were serum starved for 48 hours, fixed and stained for ARL13B (green, cilia) and either Centrin (centrioles, red; *B*–*D*) or for polyglutamylated‐tubulin with the GT335 antibody (centrioles and axoneme, red; *F*–*H*). Nuclei were stained with DAPI. (*E*) Ciliogenesis was quantified based on co‐staining with ARL13B and Centrin and expressed as % of ciliated cells (100 cells, *n* = 1).

**Fig. 4:**
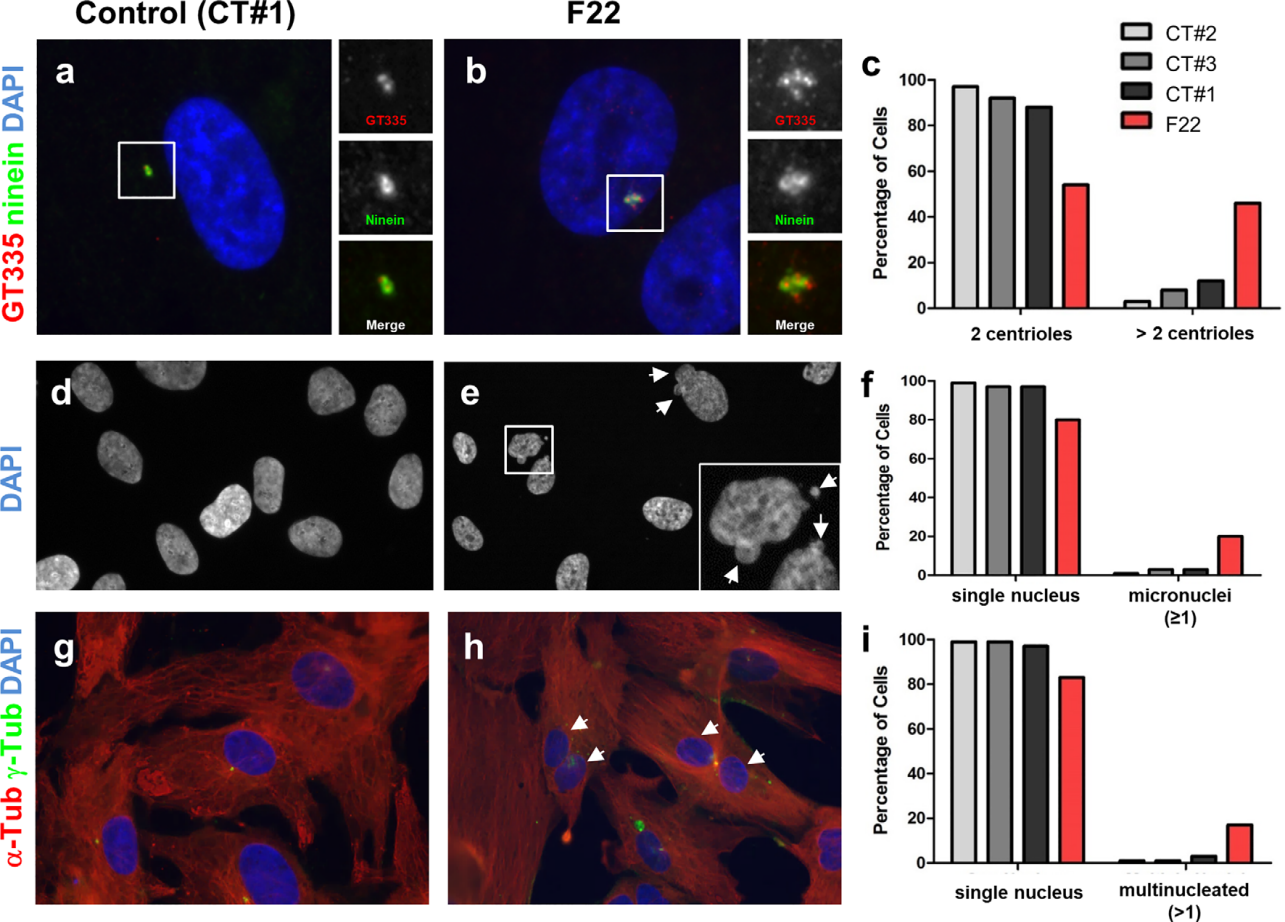
Amnioblasts from SRPS fetus showed amplification of centrioles, micronuclei and increased proportion of binucleated cells. (*A*–*C*) Cycling control (CT#1, CT#2, and CT#3) and F22 cells were stained for polyglutamylated tubulin with GT335 (red, centrioles) and for ninein (mother centrioles, green). Nuclei were stained with DAPI. The percentage of cells presenting with two or more than two centrioles (GT335) was quantified (*C*; 100 cells, *n* = 1). (*D*–*F*) DAPI staining from cells in *A* and *B* was used to identify the presence of micronuclei (white arrows). The percentage of cells with one or more micronucleus was quantified (*F*; 100 cells, *n* = 1). (*G*–*I*) Cycling control (CT#1, CT#2, and CT#3) and F22 cells were stained for α‐tubulin (red) and g‐tubulin (green) to make the identification of binucleated cells easier. Nuclei were stained with DAPI (blue). The presence of binucleated was quantified as the percentage of cells with more than one nucleus (100 cells, *n* = 1).

The motor domain is crucial for kinesin functions, including that of KIF24.^(^ ^4^ ^)^ The presence of damaging variations within this domain (Fig. 1*G*) in families 1 and 3 was therefore expected to affect its function in ciliogenesis. Although we unfortunately could not obtain fibroblasts from family 1, we did obtain material from one of the fetuses from family 3 in which we could monitor primary cilia formation (ciliogenesis) and other cellular phenotypes (see below, Figs. 3 and 4). We also obtained fibroblasts from affected individual II.1 from family 2.

Amnioblasts from the affected fetus 2 (F22) from family 3 were expanded in vitro and their ability to form PC was compared to that of cells from unrelated controls including control fetal amnioblasts (CT#1). Ciliogenesis was monitored on serum‐starved cells that were stained for ARL13B, a widely used and ubiquitous ciliary membrane marker (green), and for centrin, a centriolar marker which also stains basal bodies (red; Fig. 3*A*–*E*). Ciliogenesis was quantified as the percentage of cells harboring a PC; ie, percentage of cells presenting with a single rod like ARL13B‐stained structure associated to a centrin‐stained centriole at one of its two ends. We determined that although control cells presented a similar ciliogenesis efficiency (60% to 80% of ciliated cells), F22 fibroblasts did not ciliate properly (<5%; Fig. 3*B*,*C*,*E*). This was a very drastic effect, indicating that ciliogenesis is severely affected in cells expressing the p.Asn522Ser KIF24 variant. In contrast, ciliogenesis was not affected in cells from individual II.1 from family 2 (Fig. S1), a result in agreement with the milder skeletal phenotype.

Interestingly, in addition to the ciliogenesis defect, we observed long centrin‐positive structures in some F22 cells (Fig. 3*D*; <5% of the starved cells), whereas these were never detected in control cells (Fig. 3*B*), in which centrin staining always appeared as two small neighboring spots corresponding to the distal ends of the two centrioles. These centrin‐positive structures did not correspond to cilia because they were not stained by the ciliary membrane marker ARL13B (Fig. 3*D*). Therefore, they were likely to represent elongated centrioles, similar to those observed in some cell types in the absence of CP110, a key partner of KIF24.^(^ ^4^ ^)^ Similar results were obtained using the GT335 antibody (polyglutamylated‐tubulin), which stains both centrioles of the basal bodies and the axoneme of cilia (Fig. 3*A*). Indeed, in control cells, GT355 (red) stained the two centrioles of the basal bodies as well as the axonemes of cilia, which are also positive for ARL13B (green; Fig. 3*F*). In F22 cells (Fig. 3*G*), similarly as observed for centrin (Fig. 3*C*), most of the cells presented GT335 spots not associated with an ARL13B rod‐like staining, in agreement with a strong ciliogenesis block. Long GT335‐positive and ARL13B‐negative structures were also observed in some F22 cells (Fig. 3*H*), in agreement with the presence of elongated centrioles in some *KIF24* mutant cells. Interestingly, CP110 was still present at the tip of those elongated centrioles (Fig. S2), likely in agreement with the fact that the Asn522Ser variation did not affect interaction of KIF24 with CP110 (Fig. S3). Altogether, these results demonstrate that cells from affected fetus F22 show strong defects in ciliogenesis associated with abnormal centriole elongation.

Interestingly, *Ccp110* knockout mice (*CP110* orthologue) present skeletal dysplasia resembling Jeune/SRPS. Fibroblasts grown from these mice show a strong ciliogenesis defect as well as anomalies similar to those observed in F22 cells, including increased number of centrioles and cytokinesis defects.^(^ ^14^ ^)^ Strikingly, our analysis of centriole numbers using GT335 (red) and ninein (green) stainings indicated that more than 40% of cycling F22 cells presented more than two centrioles compared to less than 10% for control cells lines (Fig. 4*A*–*C*), in agreement with an increased number of centrosomes in *KIF24* mutant cells. In addition, micronuclei identified as small DAPI‐positive structures in the cytoplasm or in close contact with the nucleus, were present in F22 but not in control cells (Fig. 4*D*–*F*; arrows). The presence of micronuclei is due to chromosome segregation failure often caused by mitotic spindle organization/dynamic defects.^(^ ^15^ ^)^ In agreement, an increased proportion of binucleated cells were observed in *KIF24* mutant fibroblasts (Fig. 4*G*–*I*), indicating a failure in cytokinesis.

In summary, our results revealed that cells from affected F22 present not only ciliogenesis defects, but also a number of anomalies including increased number of centrosomes, presence of micronuclei, and increased proportion of binucleated cells (Figs. 3 and 4); phenotypes also found in cells lacking CP110, the main partner of KIF24.^(^ ^4^ ^)^

## Discussion

We describe novel forms of acromesomelic and spondylar skeletal dysplasia in two unrelated families and a fetal skeletal ciliopathy resembling ATD in a third family. Biallelic variants in *KIF24* were identified as the likely cause of the disorder in all affected members of these three families.

*KIF24* is located on chromosome 9p13.3 and has two annotated isoforms (http://asia.ensembl.org/Homo_sapiens/Transcript/Summary?db=core;g=ENSG00000186638;r=9:34252380-34329268;t=ENST00000402558). The longest (NM_194313.2, ENST00000402558.7) transcript is comprised of 13 exons, which encodes a 1368–amino acid protein belonging to the kinesins family. While most kinesins are molecular motors involved in the transport of cargos (proteins, lipids, organelles) along microtubules, some other kinesin family members (KIF) participate in the regulation of microtubule dynamics through their microtubule depolymerizing activity. Both types of kinesins were shown to play important roles in the assembly/disassembly cycle of primary cilia as well as in its signaling functions.^(^ ^16^ ^)^ Variants in more than 20 different kinesins have been found in phenotypically distinct autosomal dominant or recessive disorders including microcephaly and related brain anomalies, spastic paraplegia, schizophrenia, intellectual disability, retinopathies, and renal hypodysplasia.^(^ ^16^, ^17^ ^)^

Four recurrent autosomal dominant missense variants affecting two adjacent amino acids in the motor domain of another kinesin family protein encoded by *KIF22* have been identified in several patients with spondyloepimetaphyseal dysplasia with joint laxity (SEMD; MIM 603546).^(^ ^18^, ^19^ ^)^ The phenotype of all individuals harboring these variants was uniform, indicating genetic homogeneity. The phenotype of affected individuals in the families participating in the current study are heterogeneous and show a full spectrum from severe lethal skeletal dysplasia (family 3) to much milder skeletal manifestations with platyspondyly and metaphyseal dysplasia (family 2). The phenotype in family 1 is quite unique and in contrast to that observed in patients with variants in *KIF22*. Patients with *KIF24* variants in family 1 had prominent mid‐face compared to the mid‐face hypoplasia in patients with *KIF22* variants.^(^ ^18^ ^)^ They had short and broad fingers, whereas patients with *KIF22* variants have long and slender fingers. Moreover, affected individuals in family 1 had normal to wide proximal femurs in contrast to slender and tapering proximal hip in patients with *KIF22* variants.^(^ ^18^ ^)^

The identified *KIF24* variants in families 1 and 3 affect residues Ile486 and Asn522 of KIF24, which are found within the motor domain (amino acids [aa] 223–554; Fig. 1*G*). The two amino acids Ile486 and Asn522 are highly conserved among orthologues from diverse vertebrate species, indicating functional importance of these residues to KIF24. Interestingly, mutagenesis of residues 483–485 (Lys‐Glu‐Cys) to alanine resulted in decreased ability of mutant KIF24 to suppress cilia formation, although the global structure of KIF24 was not altered.^(^ ^4^ ^)^ Because the identified variant in one of our families affects residue 486, it is possible that KIF24 activity is affected in the same way as observed after mutagenesis of the nearby 483–485 (Lys‐Glu‐Cys) residues. The Asn522 residue is part of the motor domain region, which has been implicated in the interaction with NEK2 (509–547). Although a mutation in this region could be expected to affect KIF24 function in cilia disassembly through defective interaction with NEK2, our data clearly show a severe ciliogenesis defect in cells harboring this variant, suggesting a more general effect on KIF24 function.

The residues Ser566 and Thr604, mutated in family 2, are partially conserved among mammals and both affect KIF24 region just after its motor domain. The role of this domain in KIF24 function has not been characterized. Interestingly, this region corresponds to the neck domain in other KIFs, which is involved in regulatory functions. We can also speculate that the affected serine and threonine could be sites of phosphorylation, events which have been shown to regulate KIF24 functions.^(^ ^4^ ^)^ In addition, these residues are polar, hydroxyl group–containing amino acids and their alterations to nonpolar amino acids (phenylalanine and methionine, respectively) might affect KIF24 folding and/or interaction.

Interestingly our recent work on mutations in *KIF14* revealed a clear phenotype/genotype correlation with missense (or nonsense) mutations in the motor domain associated with severe lethal syndromic disease accompanied by severe microcephaly and renal hypodysplasia, whereas missense mutations in the regulatory domain C‐terminal to the motor domain led to a milder phenotype including isolated microcephaly and/or intellectual disability.^(^ ^20^ ^)^ It appears that there is a similar genotype/phenotype correlation for *KIF24* with mutations in the motor domain leading to the most severe forms in family 1 and 3, whereas mutations in family 2 affecting residues C‐terminal to the motor domain result in a less severe, yet still significantly debilitating, skeletal disorder.

Finally, analyses revealed that cells from an affected fetus harboring a homozygous missense variation in *KIF24* present defects related to ciliogenesis and cytokinesis/mitosis that are very similar to those observed in cells isolated from *Ccp110* knockout mice.^(^ ^14^ ^)^ In addition to these cellular phenotypes, the *Ccp110* knockout mice show skeletal phenotypes that are in line with the features observed in the affected fetuses in family 3. Several differences can, however, be noticed, including the presence of situs inversus and polydactyly in the mouse model not observed in the affected fetuses with *KIF24* variant. The presence of microcysts in kidneys observed in one of the affected fetuses was not described in *Ccp110* knockout mice even though ciliogenesis in the kidneys was as strongly affected as in other tissues. Despite some differences, the clinical features and the ciliary phenotypes in the cells isolated from one of the fetuses from family 3 are reminiscent of those observed in the *Ccp110* knockout mouse model and thus strongly suggest that the identified missense variations in *KIF24* lead to impaired CP110 function in both ciliogenesis and centriole amplification.

We recognize some limitations in our functional studies. These limitations are mainly linked to the fact that we could not obtain cells from affected individuals from family 1 and that we experienced severe growth problems of cells from the affected fetus 2 from family 3, which limited the number of possible investigations that could be optimized. Furthermore, we were unfortunately unable to thaw frozen batches of those cells and could not replicate experiments or investigate additional interesting questions. Finally, we could obtain fibroblasts from the affected individual in family 2, but could not detect defects in ciliogenesis in the cells, in agreement with the patient's much milder phenotype, distinct from skeletal ciliopathies.

Altogether, our data implicate missense variations in *KIF24* as a cause of a wide spectrum of skeletal ciliopathies ranging from a fetal skeletal ciliopathy to acromesomelic skeletal dysplasia and a less severe spondylometaphyseal dysplasia. Our findings add KIF24 to the list of kinesins implicated in genetic diseases (kinesinopathies)^(^ ^17^ ^)^ and provide a new interesting example of the key role of primary cilia in skeletal development.

## Author Contributions

**Louise Madeline Reilly:** Investigation. **Noor ul Ain:** Investigation. **Mari Muurinen:** Investigation. **Alice Tata:** Investigation; methodology. **Céline Huber:** Investigation. **Marleen Simon:** Investigation. **Tayyaba Ishaq:** Investigation. **Nick Shaw:** Investigation. **Wolfgang Högler:** Investigation. **Maarten F. C. M. Knapen:** Investigation. **Myrthe van der Born:** Investigation. **Sophie Saunier:** Supervision. **Sadaf Naz:** Conceptualization; investigation; supervision. **Valerie Cormier‐Daire:** Supervision; validation. **Alexandre Benmerah:** Conceptualization; supervision; validation. **Outi Makitie:** Conceptualization; investigation; supervision; validation.

## Conflicts of Interest

All authors state that they have no conflicts of interest.

### Peer Review

The peer review history for this article is available at https://publons.com/publon/10.1002/jbmr.4639.

## Supporting information


**Appendix S1**: Supplemental Materials and Methods
**Fig. S1** Ciliogenesis is not perturbed in fibroblasts from the affected individual from family 2.
**Fig. S2** CP110 is present at centrioles in F22 fibroblasts.
**Fig. S3** The N522S variation does not affect the KIF24/CP110 interaction.
**Table S1** Filtered variants identified in family 1 after analysis of WGS data.
**Table S2** Filtered variants identified in family 2 after analysis of WES data.

## Data Availability

The data supporting the results are included in the main text and in the Supplemental Material. Additional data are available from the corresponding author upon request.
