# Supplementary material for: Biallelic KIF24 Variants Are Responsible for a Spectrum of Skeletal Disorders Ranging From Lethal Skeletal Ciliopathy to Severe Acromesomelic Dysplasia
Source: J Bone Miner Res. 2022 Jul 19;37(9):1642–52. doi: 10.1002/jbmr.4639 (PMC9545074; doi:10.1002/jbmr.4639)
Supplement: Supplementary file 1 — Appendix S1: Supplemental Materials and Methods Fig. S1 Ciliogenesis is not perturbed in fibroblasts from the affected individual from family 2. Fig. S2 CP110 is present at centrioles in F22 fibroblasts. Fig. S3 The N522S variation does not affect the KIF24/CP110 interaction. Table S1 Filtered variants identified in family 1 after analysis of WGS data. Table S2 Filtered variants identified in family 2 after analysis of WES data. [file JBMR-37-1642-s001.pdf]

Supplemental material

Biallelic *KIF24* variants are responsible for a spectrum of skeletal disorders ranging from lethal skeletal ciliopathy to severe acromesomelic dysplasia

Madeline Louise Reilly[1,*], Noor ul Ain[2,3,*], Mari Muurinen[4,5,6], Alice Tata[1], Céline Huber[7,8], Marleen Simon[9], Tayyaba Ishaq[2], Nick Shaw[10,11], Salla Rusanen[4], Minna Pekkinen[4,5,6], Wolfgang Högler[11,12], Marteen F. C. M. Knapen[13], Myrthe van den Born[14], Sophie Saunier[1], Sadaf Naz[2], Valérie Cormier-Daire[7,8,&], Alexandre Benmerah[1,&] and Outi Makitie[3,4,5,6,&]

This PDF file includes:

Supplemental material and methods

Supplemental Table S1 and S2

Supplemental Figures S1 to S3

Supplemental material and methods

Plasmids and transfection of HEK 293 cells:

CP110-Flag and GFP-KIF24 encoding plasmids were described previously[1] . The Asn525Ser variation was introduced by site directed mutagenesis as described previously[2]. human embryonic kidney 293 (HEK293) were used for co-transfection experiments. Briefly, HEK293 were cultured in Dulbecco's Modified Eagle Medium (DMEM; Gibco®, Thermo Fisher Scientific) supplemented with 10% fetal bovine serum (FBS; Invitrogen, Thermo Fisher Scientific), glutamine and penicillin/streptomycin, and transiently transfected using Lipofectamine 2000 (Thermo Fisher Scientific, 11668-019).

Biochemistry

Transiently transfected HEK293 cells were lysed in 0.5% triton, 150 mm NaCl and 50 mm pH 7.5 Tris–HCl. Lysates were cleared upon incubation with mouse isotypic control antibodies and G-protein beads (Sigma-Aldrich, P7700) for 2 h at 4°C. Precleared lysates (1 mg of proteins) were immunoprecipitated using a mouse monoclonal anti-GFP antibodies (Sigma-Aldrich, 11814460001) coupled to G-protein beads for 3 h at 4°C. Beads were washed three times with increasing amounts of NaCl (150, 300 and 600 nm in 50 mm Tris–HCl pH 7.5), resuspended in 2× sample buffer (Sigma-Aldrich, S3401) and boiled at 95°C for 5 min. For immunoblotting, lysates and immunoprecipitates were separated by polyacrylamide gel electrophoresis (SDS-PAGE) and transferred onto polyvinylidene fluoride transfer membranes (GE Healthcare). Immunoblotting was performed using rabbit anti-GFP (Invitrogen, A11122) and mouse anti-CP110 (Proteintech, 12780-1-AP) antibodies and revealed using the ECL+ Detection Kit (GE Healthcare).

References

1. Kobayashi T, Tsang WY, Li J, Lane W, Dynlacht BD. Centriolar kinesin Kif24 interacts with CP110 to remodel microtubules and regulate ciliogenesis. Cell. 2011;145(6):914-25.

2. Bizet AA, Becker-Heck A, Ryan R, et al. Mutations in TRAF3IP1/IFT54 reveal a new role for IFT proteins in microtubule stabilization. Nat Commun. 2015;6:8666.

Supplementary Material

Table S1: Filtered variants identified in family 1 after analysis of WGS data.

Chr	Position	Ref	Alt	Gene	Transcript	Polyphen Prediction	SIFT Prediction	CADD Score	EXAC-Frequency	gnomAD-Frequency
2	163027538	T	A	FAP	ENST00000188790	0.236/B	T	22.8	2.47E-05	3.66E-05
3	52555641	G	A	STAB1	ENST00000321725	0.523/PD	T	24.1	3.30E-05	2.87E-05
7	143632672	C	T	OR2F2	ENST00000408955	0.213/B	D	13.69	2.47E-05	1.62E-05
9	34263158	T	C	KIF24	ENST00000345050	0.001/B	D	26.3	4.14E-05	3.27E-05
9	36923412	G	C	PAX5	ENST00000358127	0.034/B	T	19.03	1.65E-05	2.04E-05
11	19901540	G	A	NAV2	ENST00000349880	0.121/B	T	16.32	Absent	4.08E-06
11	47603688	A	G	NDUFS3	ENST00000263774	0.967/PD	D	32	Absent	Absent
11	62488793	T	C	HNRNPUL2	ENST00000301785	0.047/B	T	22.5	2.48E-05	2.09E-05
12	56094794	G	A	ITGA7	ENST00000257879	0.953/PD	D	33	8.24E-06	1.62E-05
12	57389345	A	T	GPR182	ENST00000300098	0.85/PD	D	22.6	8.24E-06	8.13E-06
12	58000762	G	A	DTX3	ENST00000337737	0.006/B	T	22.7	0.0001489	0.000114
15	33360028	C	G	FMN1	ENST00000334528	0	D	13.87	2.48E-05	2.45E-05

*Positions are with reference to GRCh37/hg19.

Table S2: Filtered variants identified in family 2 after analysis of WES data.
*Positions are with reference to GRCh37/hg19.

Type	Chr	Position	Ref	Alt	Gene	Transcript and description of variant	Polyphen2 Prediction (HDIV/HVAR)	SIFT Prediction	CADD Score	gnomAD Exome Frequency
De novo	1	233515268	G	C	MAP3K21	NM_032435:c.2516G>C:p.C839S	B/B	T	0.002	absent
De novo	7	143807517	A	T	OR2A2	NM_001005480:c.842A>T:p.N281I	P/P	D	23.0	absent
X-chromosomal	X	41586847	C	T	GPR82	NM_080817:c.568C>T:p.R190W	B/B	D	17.37	absent
X-chromosomal	X	57618915	G	A	ZXDB	NM_007157:c.434G>A:p.R145H	B/B	T	4.802	0.0002
Compound heterozygous	1	17318873<br>17332000	C<br>C	T<br>T	ATP13A2	NM_001141973:c.1855G>A:p.V619I<br>NM_001141973:c.157G>A:p.V53M	B/B<br>B/B	T<br>T	0.959<br>19.57	6.139e-05<br>6.115e-05
Compound heterozygous	2	109100601<br>109100770	G<br>G	T<br>A	GCC2	NM_181453:c.3451-4G>T<br>NM_181453:c.3613+3G>A	na<br>na	na<br>na	na<br>na	0.0002<br>0.0015
Compound heterozygous	9	34257794<br>34257908	G<br>G	A<br>A	KIF24	NM_194313:c.1811C>T:p.T604M<br>NM_194313:c.1697C>T:p.S566F	P/B<br>P/B	T<br>T	12.02<br>20.9	0.0002<br>0.0057
Compound heterozygous	11	62287107<br>62293817<br>62294810	G<br>T<br>A	C<br>C<br>C	AHNAK	NM_001346445c.14782C>G:p.P4928A<br>NM_001346445:c.8072A>G:p.K2691R<br>NM_001346445:c.7079T>G:p.V2360G	B/B<br>D/D<br>D/D	T<br>D<br>D	22.6<br>19.08<br>20.0	0.0005<br>0.0005<br>1.219e-05
Compound heterozygous	19	50393043<br>50394693	C<br>G	T<br>A	IL4I1	NM_152899:c.1588G>A:p.V530M<br>NM_152899:c.605C>T:p.A202V	P/P<br>P/B	D<br>T	11.0<br>12.42	0.0065<br>0.0025
Compound heterozygous	20	42543527<br>42635391	C<br>C	T<br>A	TOX2	NM_001098797:c.-5C>T<br>NM_001098797:c.370C>A:p.L124I	na<br>D/D	na<br>D	na<br>24.4	0<br>0.0020

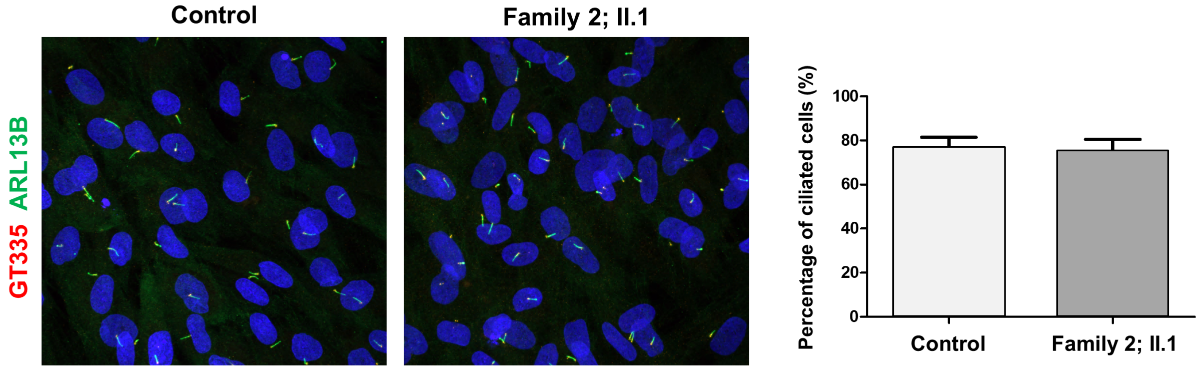

Supplemental Figure S1: Ciliogenesis is not perturbed in fibroblasts from the affected individual from family 2.

Fibroblasts from age-matched control and from the II.1 individual from family 2 were serum starved for 24 hours, fixed and stained with antibodies against ARL13B (green, cilia) and GT335 (basal body, red). Nuclei were stained with DAPI (blue). Ciliogenesis (percentage of ciliated cells) was quantified based on those stainings. n=3 independent experiments.

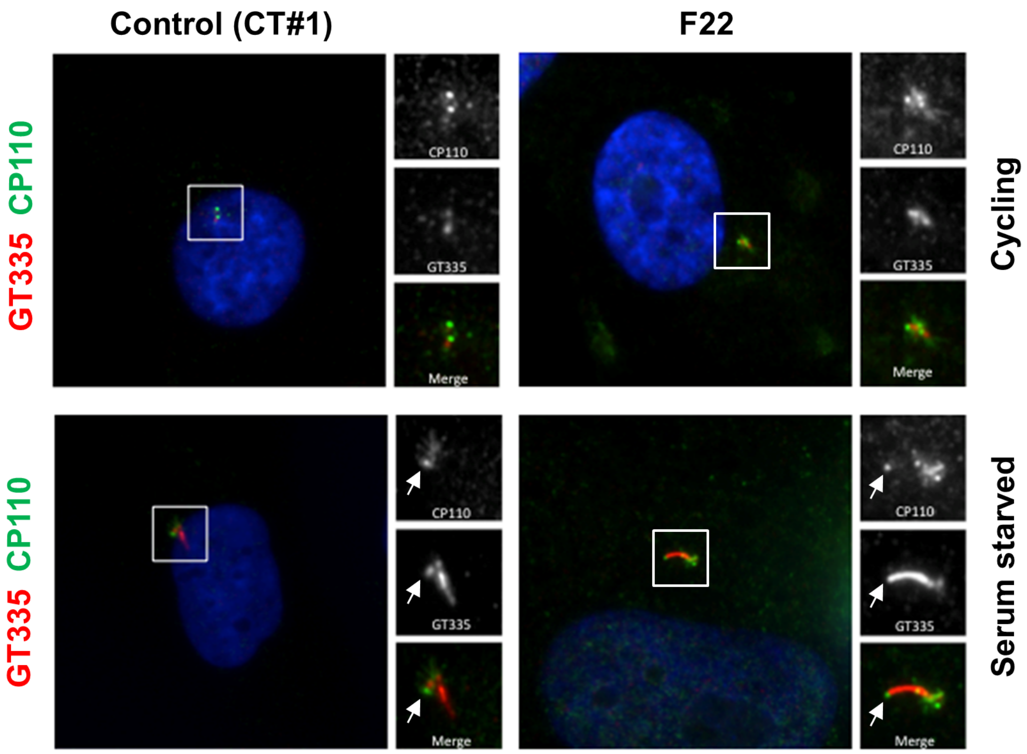

Supplemental Figure S2: CP110 is present at centrioles in F22 fibroblasts.

Cycling or serum-starved (48 hours) control and F22 fibroblasts were fixed and stained with anti-CP110 (green) and GT335 (centrioles and cilia, red) antibodies. Nuclei were stained with DAPI (blue). White boxes indicate zoomed regions which are depicted on the right. White arrows stress for centriolar CP110 dots.

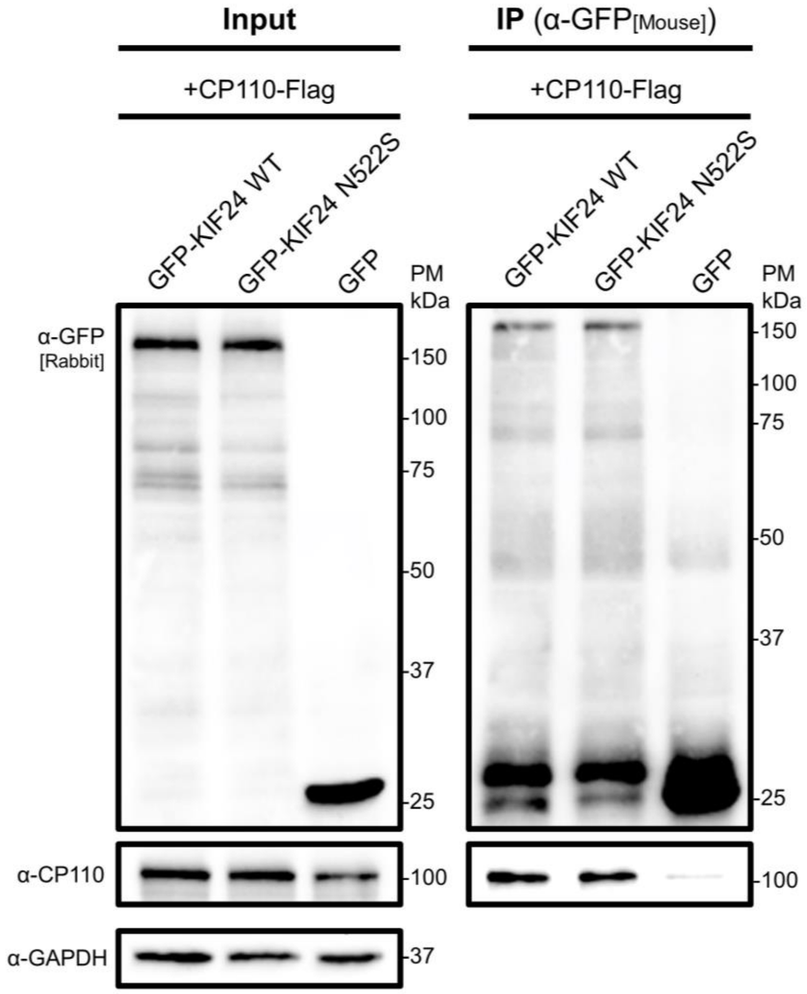

Supplemental Figure S3: The N522S variation does not affect the KIF24/CP110 interaction.

HEK293 cells were co-transfected with indicated plasmidic constructs, lysed and immunoprecipitated with an anti-GFP antibody. Lysates (input) and immunoprecipitates (IP) were analyzed by western-blotting using anti-GFP and anti-CP110 and anti-GAPDH antibodies as indicated. n=2, one representative experiment is shown.
